# Phenotypic Variability of *MEGF10* Variants Causing Congenital Myopathy: Report of Two Unrelated Patients from a Highly Consanguineous Population

**DOI:** 10.3390/genes12111783

**Published:** 2021-11-10

**Authors:** Mohammad AlMuhaizea, Omar Dabbagh, Hanan AlQudairy, Aljouhra AlHargan, Wafa Alotaibi, Ruba Sami, Rahaf AlOtaibi, Mariam Mahmoud Ali, Hindi AlHindi, Dilek Colak, Namik Kaya

**Affiliations:** 1 Neuroscience Center, King Faisal Specialist Hospital and Research Center, Riyadh 11211, Saudi Arabia; mmuhaizea@kfshrc.edu.sa (M.A.); ODABBAGH@kfshrc.edu.sa (O.D.); wafaasattam@gmail.com (W.A.); ruba.sami@hotmail.com (R.S.); rahoofa788@gmail.com (R.A.); dr.mariam999@gmail.com (M.M.A.); 2 Department of Translational Genomics, Center for Genomic Medicine, KFSHRC, Riyadh 11211, Saudi Arabia; halqudairy@kfshrc.edu.sa (H.A.); aljouhra.hargan@gmail.com (A.A.); 3 Department of Pathology and Laboratory Medicine, KFSHRC, Riyadh 11211, Saudi Arabia; HAL-hindi@kfshrc.edu.sa; 4 Department of Biostatistics, Epidemiology and Scientific Computing, KFSHRC, Riyadh 11211, Saudi Arabia; dkcolak@gmail.com

**Keywords:** *MEGF10*, congenital myopathy, novel variants, splicing, convex scoliosis, butterfly vertebrae, atelectasis, bronchiectasis, flexion deformity, subluxation

## Abstract

Congenital myopathies are rare neuromuscular hereditary disorders that manifest at birth or during infancy and usually appear with muscle weakness and hypotonia. One of such disorders, early-onset myopathy, areflexia, respiratory distress, and dysphagia (EMARDD, OMIM: 614399, MIM: 612453), is a rare autosomal recessive disorder caused by biallelic mutations (at homozygous or compound heterozygous status) in *MEGF10* (multiple epidermal growth factor-like domains protein family). Here, we report two unrelated patients, who were born to consanguineous parents, having two novel *MEGF10* deleterious variants. Interestingly, the presence of *MEGF10* associated EMARDD has not been reported in Saudi Arabia, a highly consanguineous population. Moreover, both variants lead to a different phenotypic onset of mild and severe types. Our work expands phenotypic features of the disease and provides an opportunity for genetic counseling to the inflicted families.

## 1. Introduction

Congenital myopathies (CMs) are rare inherited conditions with a broad phenotypic and genetic diversity. CMs manifest after birth or during infancy with static or slowly progressive clinical course [1]. Despite their phenotypic diversity, patients demonstrate common symptoms, including hypotonia, muscle weakness, dysmorphic features, and respiratory insufficiency [2,3,4]. Based on the histological features of muscle biopsy, CMs are classified into five main types: nemaline myopathies, core myopathies, centronuclear myopathies (CNM), congenital fiber-type disproportion, and myosin storage myopathies, where each is further divided into different subtypes [4,5]. Rod-like inclusions in muscle biopsy characterize nemaline myopathies while cores in muscle biopsy distinguish core myopathies from the other types. Central core disease (CCD) and multiminicore disease (MmD) (core myopathies) are the most common form of congenital myopathies. Various mutations in 27 different genes have been linked to CMs and they may cause the same disease phenotype [1,4,6]. On the other hand, different mutations in the same gene may result in various types of CMs [7].

In 2011, Logan et al., reported the first *MEGF10* mutation associated with CMs [8]. *MEGF10* encodes a transmembrane receptor belong to multiple epidermal growth factor-like domains protein family. It is expressed in the central nervous system (CNS) predominantly in the brain, astrocytes, and satellite cells of skeletal muscle. The receptor has a critical role in mediating apoptosis during cell phagocytosis by binding to phosphatidylserine expressed on the surface of apoptotic cells [9]; hence, it involves in cell adhesion, motility, proliferation, and phagocytosis through macrophages and astrocytes of apoptotic cells. Satellite cells work as a precursor of muscle cells. In normal cases, satellite cells are inactive in resting muscles. During exercise, trauma, or muscle injury, these cells become activated and *MEGF10* is upregulated to produce myogenic cells, which differentiate into new muscle fibers and fuse with existing fibers. This explains satellite cells’ depletion in *MEGF10* associated congenital myopathies [8]. *MEGF10* that is located on the long arm of chromosome 5q23.2. Currently, there are several reported mutations in *MEGF10* including small deletions and insertions, various missense, nonsense, splicing, and a large gross deletion that expands 757 base pairs spanning over exon 7. These mutations have been reported to cause autosomal recessive congenital myopathy, areflexia, respiratory distress, and dysphagia with early or late-onset syndrome [10,11] (abbreviated as EMARDD, OMIM: 614399, MIM: 612453), minicore myopathy [9,12,13], and limb girdle muscular dystrophy [14,15], and muscle weakness [16]. Missense, frameshift indels, and nonsense mutations in *MEGF10* cause respiratory distress usually induced by diaphragmatic paralysis. Affected individuals frequently become ventilator dependent or die secondary to respiratory failure [17].

This study reports two unrelated Saudi patients with mild and severe congenital myopathy due to two different novel variants in *MEGF10*. These are the first two patients to be reported in Saudi Arabia, reflecting the possibility of under-recognized cases in the Gulf region, where autosomal recessive conditions prevail due to the consanguinity [18].

## 2. Materials and Methods

### 2.1. Patients and Ethics

**Figure 1:**
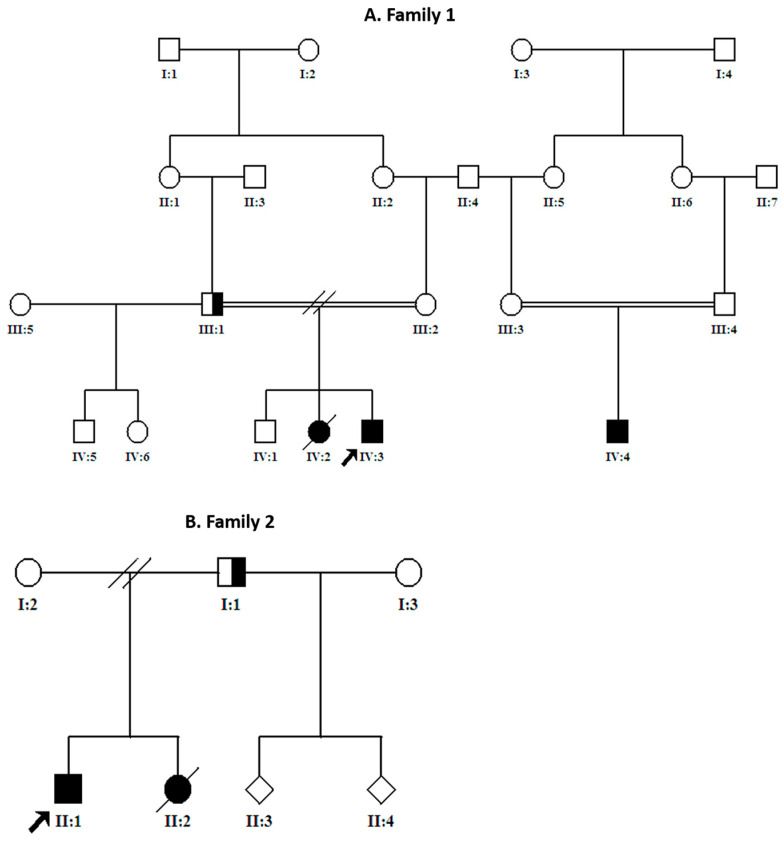
Family pedigrees indicating affected members. (**A**) Extended pedigree of the first family shows two branches of Table 2. IV3, and IV4). Among the patients, only the index case was genetically tested. (**B**) Pedigree of the second family shows two affected siblings in the family.

Two boys from two separate unrelated families were included to the project. The parents in each family are first-degree cousins. The patients were presented to our institution (King Faisal Specialist Hospital and Research Center, KFSHRC) with variable degrees of hypotonia and weakness for investigation and carefully examined by board-certified pediatric neurologists at the neuroscience clinics in the hospital (Table 1) family pedigrees are presented in Figure 1. After obtaining the signed informed consents (approved by institutional review board, Research Advisory Council at Office of Research Affairs at KFSRCH, RAC#2120022), blood samples (5 mL for each individual) from the patients and unaffected family members were collected into EDTA tubes. To establish EBV transformed lymphoblastoid cell lines, additional blood samples from probands were collected into heparin tubes (3 mL).

**Table 1:** Patients’ clinical and genetic features.

Patients	Patient 1	Patient 2
Muscle Biopsy	Unremarkable.	Not done
Clinical Features	Plagiocephaly, forehead ridge and hirsutism, down slanting eyes, epicanthal folds, mild bilateral ptosis, symmetric facial weakness, low set ears with backward rotation, high arched palate, long fingers, camptodactyly of ring and middle fingers, pectus excavatus, low hair line, sacral dimple in the lower back and scoliosis, areflexia	Plagiocephaly, symmetric myopathic facies, high arched palate, low set ears, scoliosis, generalized hypotonia, areflexia
Outcome	Death due to respiratory infection.	Survival (patient is currently alive, last visit was at age of 9 years old)
Brain MRI	Mild bilateral frontalatrophic changes	Not done
EMG	Myopathic changes in tibialis anterior and vastus lateralis muscles	Neurogenic changes
Genotype	*MEGF10*:c.3132dupA:p.Glu1045Argfs*22:homozygous	NM-032446.2:c.2980+5 G>C:Splice site variant, homozygous
O_2_ req.	NC., 1-2L	None
Dysphagia	Gastronomy tube feeding (Severe dysphagia)	Oral (Mild oral dysphagia)
Best motor ability	Non-sitting	Ambulant
Onset of Symptoms	Since birth	Since birth

### 2.2. DNA Extraction

DNA isolation was carried out using commercially available kits according to the manufacturer’s instruction (Gentra Systems, Minneapolis, MN, USA). DNA quality and quantity were checked using Nanodrop instrument (ND-1000) (Thermo Fisher Scientific Corp., Waltham, MA, USA) and stored at −20 °C for further use such as next generation sequencing and Sanger sequencing reactions.

### 2.3. Gene Panel Screening, Variant Detection, and Sanger Sequencing

A comprehensive gene panel consisting of numerous genes and mutations (Supplementary Table S1) were designed and used on Ion Torrent Proton sequencing platform according to manufacturer’s guidelines (Thermo Fisher Scientific Corp.). Briefly, the reads were processed by the Torrent Suite Software and variant annotation web server using Ion Reporter Software (Thermo Fisher Scientific Corp.). Identified variants filtered and confirmed by Sanger Sequencing according to standard protocols.

### 2.4. RT-PCR

To investigate the impact of the splice site variant on the transcript, RNA extracted from the lymphoblastoid cell lines from the patient 2. First, cDNA was synthesized using High-Capacity cDNA Reverse Transcription Kit (Thermo Fisher Scientific Corp.) using random hexamers. Then *MEGF10*-specific primers designed to include the variant site and likely skipped regions were utilized during the amplification of the cDNA. RT-PCR products were analyzed on 2% agarose gel.

## 3. Results

### 3.1. Clinical Findings

#### 3.1.1. Patient 1 (Family 1)

A consanguineous Saudi family with a six-months-old boy was referred to our institution (KFHSRC) from a local hospital. The boy was suffering from congenital progressive hypotonia with a significant family history. He has two older siblings, a healthy five-year-old brother and a sister with a similar disease presentation who died at 11 months of age. The family history was free from any other CNS or metabolic disorders. The index patient was the product of a full-term pregnancy that was associated with decreased fetal movements and normal amniotic fluids. He was delivered by cesarean section due to breech presentation with a birth weight of 2.9 kg. Due to significant hypotonia, dysmorphic features, he was hospitalized at a local rural hospital for 17 days. Early in the neonatal period, his mother noted abnormal movements in the form of jerky movements of upper limbs and lower jaw lasting several minutes with no associated up-rolling of eyes nor change in level of consciousness. A dramatic response to phenobarbital was observed with complete resolution of the seizures. An electroencephalogram (EEG) at four months of age at his local hospital revealed generalized epileptiform discharges. A follow up EEG at 6 months of age revealed complete resolution of these discharges. He had two chest infections requiring hospital admission.

The six-month-old child showed developmental delay upon examination, and his growth parameters were at a rate below the 3rd percentile (weight: 3.7 kg, length: 57 cm, and head circumference: 38.5 cm). He was dysmorphic with evidence of plagiocephaly, forehead ridging, and hirsutism with down slanting eyes, epicanthal folds, mild bilateral ptosis, facial diplegia, low set posteriorly rotated ears, high arched palate, low hairline, long fingers, camptodactyly of the ring and middle fingers, pectus excavatum, and a sacral dimple in the lower back with a hair tuft and scoliosis. The tongue was in the midline with no evidence of fasciculations. Severe hypotonia was noted, with significant head lag and inability to sit while supported. His deep tendon reflexes were absent. He had a weak cry and cough. His general examination revealed no skin, cardiovascular, or genital abnormalities or organomegaly. He had a good social smile. He had good regard for his own hands. He could reach and transfer objects. His extraocular movement was normal, with equally reactive pupils.

**Figure 2:**
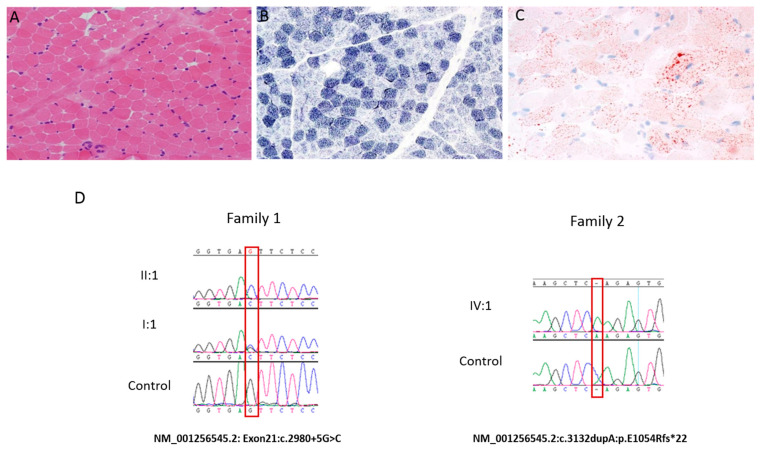
Histopathological and genetic findings. (**A**) H&E: Rare small fibers. (**B**) NADH: increased subsarcolemmal activity in some fibers. (**C**): Oil Red O stain: increase in intracellular lipid. (**D**) The image displays the chromatogram of the index patients (IV: 3 and II: 1 in each family) harboring the variants (c.3132dupA), (c.2980+5G>C), respectively.

Spinal Magnetic resonance imaging (MRI) revealed scoliosis and right diaphragmatic eventration. At age of four months, a brain MRI was carried out and showed mild bifrontal atrophy changes. A skeletal muscle biopsy of the right thigh was essentially normal and showed minimal variation in myofiber size (Figure 2A) and unremarkable endomysial connective tissue. There were no inclusions or vacuoles, and the intermyofibrillar network is intact. There was only a mild nonspecific subsarcolemmal mitochondrial proliferation (Figure 2B) and a mild increase in stainable lipids (Figure 2C). Unfortunately, the patient passed away at the age of eight months secondary to respiratory failure following a severe chest infection. His affected sister presented to a local hospital with early life severe hypotonia, muscle weakness, dysphagia, oxygen dependency, and dysmorphic features, but no definite diagnosis was established at the time. She died at the age of 11 months due to respiratory failure.

**Figure 3:**
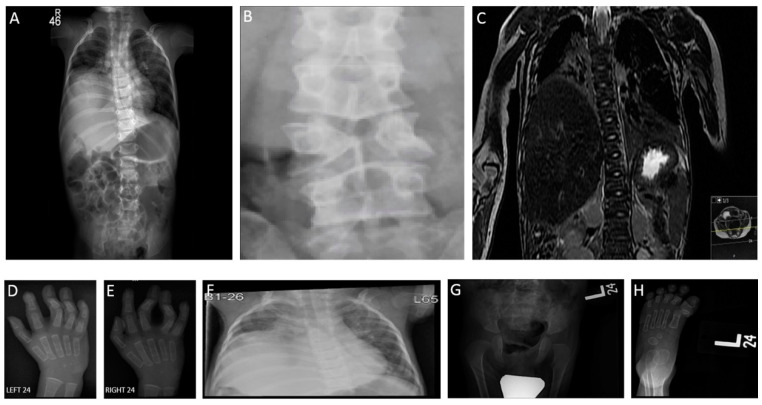
Radiological demonstration of deformities in patient 1. (**A**) Mild right convex scoliosis in the dorsal spine. (**B**) Butterfly vertebrae in the dorsal and lumber vertebra. (**C**) Mild right convex scoliosis in the dorsal spine. (**D**,**E**) Flexion deformity in the fingers especially the ring and middle fingers of both hands involve the proximal interphalangeal joints. (**F**) Elevation of the right hemidiaphragm is noted with atelectasis in the right lower lobe and bronchiectasis in both lower lobes. Heart size is normal (**G**) Subluxation of the left hip joint. (**H**) Hypoplasia of the proximal phalanx of the left big toe with subluxation of the first metatarsophalangeal joint on the left side. Very small muscles in both lower limbs. Bilateral geno valgum more on the left side with flexion of the left knee joint.

Nerve conduction study (NCS) was unremarkable. Electromyography showed myopathic changes in the tibialis anterior and vastus lateralis muscles. His skeletal survey revealed mild right convex scoliosis in the dorsal spine and butterfly vertebrae in the dorsal and lumber vertebra (Figure 3A–C). Flexion deformity in the fingers is noted, especially the ring and middle fingers of both hands involves the proximal interphalangeal joints (Figure 3D,E). Elevation of the right hemidiaphragm is noted with atelectasis in the right lower lobe and bronchiectasis in both lower lobes with average heart size (Figure 3F). There is left developmental dysplasia of the hip (Figure 3G) and hypoplasia of the proximal phalanx of the left big toe with subluxation of the left first metatarsophalangeal joints on the left side (Figure 3H). Renal ultrasound revealed bilateral nephrolithiasis.

#### 3.1.2. Patient 2 (Family 2)

A four-year-old boy from a consanguineous Saudi family (first-degree cousins) presented with neonatal-onset generalized hypotonia. The pregnancy was complicated by decreased fetal movements. He had one sister with a similar phenotype who passed away at the age of 18 months with respiratory infections. His two paternal half-siblings are healthy. The patient suffered from feeding difficulties, intermittent choking, and recurrent aspiration pneumonia. In addition, significant motor developmental delay was evident early on. At 21 months, he achieved good head control, was able to sit without support, and started to crawl and reach for objects. His last clinical follow up was at the age of 9 years when he was noted to be walking independently, but was a slow runner. He was able to draw a line and completely undress himself. He engaged in-group play and had no major cognitive deficits. He did not require long-term oxygen support.

The patient was conscious and interactive during the examination. His vital signs were stable, and the oxygen concentration was well maintained on room air. He had dysmorphic features in the form of plagiocephaly, facial diplegia, high arched palate, low set posteriorly rotated ears, mild dextro convexity scoliosis of the thoracic spine with spine rigidity. Generalized hypotonia, axial more than appendicular, as well as generalized weakness (proximal more than distal) were noted. He had a positive Gower sign but no calf muscle hypertrophy. His deep tendon reflexes were absent. He also had joint hyperlaxity of knees, wrists, and elbows. A detailed neurometabolic workup, inclusive of blood lactic and pyruvic acids, ammonia, biotinidase, tandem mass spectroscopy, urine gas chromatography and mass spectroscopy, creatine kinase, as well as thyroid function, were all normal. Nerve conduction studies (NCS) was unremarkable while EMG of the vastus lateralis muscle showed neurogenic changes more suggestive of motor neuron disease. A modified barium study showed mild oral dysphagia. Echocardiogram showed trivial pulmonary valve regurgitation with normal biventricular systolic function.

### 3.2. Genetic Analysis

**Figure 4:**
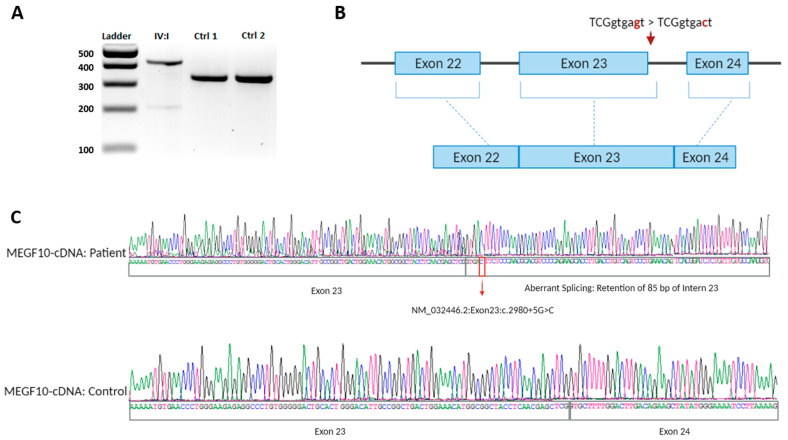
RT-PCR result for patient 2. RT-PCR was performed on RNA extracted from lymphocytic cell culture line from the patient 2 followed by 2% agarose gel electrophoresis. (**A**) Compared to both controls, the patient has a higher size band implicating presence of an aberrant transcript. (**B**) Illustration presents the predicted splicing effect. (**C**) A variant at the fifth nucleotide in intron 23 was found by DNA Sanger sequencing that is also indicated presence of a retained DNA (85 bp) from intron 23.

Gene testing for spinal muscular atrophy was negative for the patient 1. High-resolution cytogenetic evaluation based on SNP arrays did not detect any definite chromosomal abnormality or pathogenic copy number variant (CNV). A comprehensive myopathy gene panel for patient 1 revealed a homozygous variant in *MEGF10* (NM_032446.2:c.3132dupA:p.Glu1045Argfs*22) (Figure 2D). DNA analysis for patient 2 for myotonic dystrophy (*DMPK*) was unremarkable. For patient 2, comprehensive muscular dystrophy and myopathy gene panel revealed a splicing variant in *MEGF10* (NM_032446.2:c.2980+5G>C:Exon21:Chr5:126784919) (Figure 2D and Figure 4A–C). In addition to this biallelic splice site variant, the panel revealed another pathogenic variant of *PRKAG3* (c.364dupA, p.Thr122Asnfs*11) at heteroallelic state.

RT-PCR on patient’s 2 RNA revealed presence of a larger fragment in the patient’s sample in comparison to those of the controls (Figure 4A). The larger fragment was purified from the gel. Sanger sequencing analysis of the abnormal RT-PCR product showed retention of 85 bp from the intron 23, resulting in a larger aberrant transcript (Figure 4C).

## 4. Discussion

EMARDD is considered an autosomal recessive congenital myopathy characterized by early-onset at birth, or during infancy. The disease is characterized by areflexia and respiratory distress caused by diaphragmatic weakness or paralysis [1]. With progression of the disease, respiratory failure necessitates long term respiratory support, with subsequent demise of nearly half of the patients. Additional features include dysphagia often requiring gastrostomy feeding, generalized muscle weakness causing delayed motor development, poor head control, facial weakness, cleft palate, contractures, and scoliosis [12,19]. In severe cases, most patients never achieve walking [13,14]. EMARDD and MmD share some phenotypic features. There are several mutations reported in *MEGF10* that causes different onset (Table 2). Only a few reported MmD cases were caused by a *MEGF10* mutation. However, half of the reported MmD cases are caused by mutations in *SEPN1* and *RYR1* genes [12]. Moreover, MmD caused by *MEGF10* mutations is considered a mild form of EMARDD with early or late onset of symptoms [13]. All *MEGF10*-related MmD cases are caused by compound heterozygous missense mutations except for two cases. One had a single heterozygous missense (c.211C>T) and the other had splicing mutation (c.2981-2A>G). This can explain late onset of MmD in some of the cases where residual *MEGF10* prevents the development of early-onset respiratory muscle weakness [12]. Most EMARDD patients had homozygous nonsense, missense mutations, and small indels except for one patient who had a compound heterozygous mutation (a missense change (c.2320T>C), and a small deletion (c.1325delC). Therefore, it is reasonable to think that in most cases EMARDD is linked to stop codon mutations [8,12,13,20].

Here, we describe two pediatric patients in details. Both presented with hypotonia, dysmorphic features, and absent deep tendon reflexes. The 6-month-old boy had a severe phenotype presentation that caused several facial and skeletal features to be altered. As with most of the reported cases, he passed away in the first year of life due to respiratory failure. The 4-year-old boy presented with a milder phenotype. He is alive and his symptoms are milder, including dysphagia, scoliosis, and slower physical abilities compared to his peers. His phenotype is more of axial predominant myopathy with scoliosis and spine rigidity. Unfortunately, muscle histopathology could not be obtained from the milder phenotype to compare to the severe phenotype and correlated this with the EMG variability found in both cases.

Though most of the affected patients were symptomatic during early childhood, there are reports of adult presentations, who survived till their early sixties. The older cases represented a milder phenotype characterized by juvenile-onset scoliosis likely due to axial myopathy, adult-onset respiratory insufficiency, and limb muscle weakness. Joint hyperlaxity was also observed [1,12,13]. While most muscle biopsies of EMARDD patients show fiber size variation and adipose tissue infiltration [8,13], muscle biopsy of patient 1 showed minimal changes in fiber size.

The phenotype of EMARDD, particularly the early onset of diaphragmatic paralysis, is similar to spinal muscular atrophy with respiratory distress type (SMARD) [25]. The SMARD is an autosomal recessive disease caused by a mutation in the immunoglobulin μ-binding protein 2 gene (*IGHMBP2*) and *SMN1* [25,26]. It is a form of spinal muscular atrophy with phenotypic and genetic heterogeneity. The main difference between EMARDD and SMARD is the nervous system involvement. EMARDD has mainly myopathic features whereas SMARD is a neuromyopathic disease where patients suffer from the degeneration of the peripheral nerves, including the sensory and autonomic nerves, and frequently the distal muscles are the most affected. Therefore, it is desirable to make accurate differential diagnoses between various forms of SMARD and other congenital neuromuscular conditions presenting with diaphragmatic weakness [8]. As the disease varies in its presentation from milder to more severe forms, awareness of the phenotypes must be raised. This includes family counseling since consanguineous unions play a role in recessive genetic mutations manifestations [5,11,27].

## 5. Conclusions

In conclusion, this report is the first to describe the clinical presentation of two patients with two novel variants in *MEGF10* in Saudi Arabia. The phenotypic similarities between EMARDD and other congenital neuromuscular disorders such as SMARD may cause difficulties in reaching a definite diagnosis in the severe phenotype while the milder phenotype may be similar to axial predominant myopathies. Screening for dysphagia, diaphragmatic weakness and respiratory hypoventilation are important elements particularly in the milder phenotype.

## Figures and Tables

**Table 2:** List of previously reported pathogenic variants in *MEGF10* found in the literature.

No.	Variant Name (cDNA)	Variant Type	Variant Name (Amino Acid)	Phenotypic Details	Ethnicity	References
1	c.211C>T	Missense	p.R71W	Minicore myopathy	Portuguese	Boyden (2012) Neurogenetics 13, 115 [9]
2	c.230G>A	Missense	p.R77Q	Minicore myopathy	French-F/German-M	Liewluck (2016) Muscle Nerve 53, 984 [12]
3	c.352T>C	Missense	p.C118R	Muscular dystrophy, limb girdle	Unknown	Harris (2017) Orphanet J Rare Dis 12, 151 [15]
4	c.352T>C	Missense	p.C118R	Muscular dystrophy, limb girdle	Unknown	Harris (2018) Neuromuscul Disord 28: 48 [14]
5	c.976T>C	Missense	p.C326R	Minicore myopathy	Mixed European Origin	Boyden (2012) Neurogenetics 13, 115 [9]
6	c.1559G>A	Nonsense	p.W520*	EMARDD	Sri Lankan	Logan (2011) Nat Genet 43, 1189 [8]
7	c.1833T>G	Missense	p.C611W	Minicore myopathy	French-F/German-M	Liewluck (2016) Muscle Nerve 53, 984 [12]
8	c.2211G>A	Nonsense	p.W737*	Muscle weakness	Canadian/Not Specified	Wu (2018) Can J Neurol Sci epub, epub [16]
9	c.2301C>A	Nonsense	p.C767*	EMARDD	Qatari	Logan (2011) Nat Genet 43, 1189 [8]
10	c.2320T>C	Missense	p.C774R	EMARDD	English	Logan (2011) Nat Genet 43, 1189 [8]
11	c.2320T>C	Missense	p.C774R	EMARDD	Mixed European ancestry	Boyden (2012) Neurogenetics 13: 115 [9]
12	c.2429G>A	Missense	p.C810Y	Minicore myopathy	Japanese	Takayama (2016) Neuromuscul Disord 26, 604 [13]
13	c.3144T>G	Nonsense	p.Y1048*	EMARDD	Turkish	Logan (2011) Nat Genet 43, 1189 [8]
14	c.1426+1G>T	Splicing error	—	Muscular dystrophy, limb girdle	Unknown	Harris (2017) Orphanet J Rare Dis 12, 151 [15]
15	c.1426+1G>T	Splicing error	—	Muscular dystrophy, limb girdle	Unknown	Harris (2018) Neuromuscul Disord 28: 48 [14]
16	c.2981-2A>G	Splicing error	—	Minicore myopathy	Japanese	Takayama (2016) Neuromuscul Disord 26, 604 [13]
17	c.2980+5G>C	Splicing error	—	EMARDD	Saudi	THIS STUDY
18	c.131_132delTG	Small deletion	—	EMARDD	Japanese	Takayama (2014) Neuromuscul Disord 24 848 [21]
19	c.131_132delTG	Small deletion	—	EMARDD	Japanese	Takayama (2016) Neuromuscul Disord 26: 604 [13]
20	c.1325delC	Small deletion	p.Pro442Hfs*9	EMARDD	English	Logan (2011) Nat Genet 43, 1189 [8]
21	c.1557delA	Small deletion	p.Trp520fs*	Myopathy, areflexia, respiratory distress, and dysphagia	Unkown (M+P)	Posey (2017) N Engl J Med 376, 21 [22]
22	c.1557delA	Small deletion	p.Trp520fs*	Myopathy, areflexia, respiratory distress, and dysphagia	Emarati	Alabdullatif (2017) Clin Genet 91: 616 [23]
23	c.2288_2297dup10	Small insertion	p.Asp766EfsX4	EMARDD	Pakistani	Logan (2011) Nat Genet 43, 1189 [23]
24	c.3132dupA	Small insertion	—	EMARDD	Saudi	THIS STUDY
25	N/A	Large deletion (757 bp in exon 7)	—	EMARDD	Arab	Pierson (2013) Neuromuscul Disord 23, 483 [20]
26	c.2320T>C	Missense	p.C774R	EMARDD	Unknown	Saha et al, Hum Mol Genet 2019 [24]
27	c.918-2A>G	Splicing error	—	EMARDD	Unknown	Saha et al, Hum Mol Genet 2019 [24]
28	c.976T>C	Missense	p.C326R	EMARDD	Unknown	Saha et al, Hum Mol Genet 2019 [24]
29	c.211C>T	Missense	p.T1030C	*MEGF10* myopathy, adult onset	Unknown	Saha et al, Hum Mol Genet 2019 [24]

## Data Availability

Not applicable.

## Supplementary Materials

Supplementary Table. List of all genes included in the comprehensive muscular dystrophy and myopathy gene panel used to identify the pathogenic variant is available online at https://www.mdpi.com/article/10.3390/genes12111783/s1, Table S1: List of previously reported pathogenic variants in *MEGF10.*
